# Lupus nephritis correlates with B cell interferon-β, anti-Smith, and anti-DNA: a retrospective study

**DOI:** 10.1186/s13075-022-02766-1

**Published:** 2022-04-18

**Authors:** Fatima Alduraibi, Huma Fatima, Jennie A. Hamilton, W. Winn. Chatham, Hui-Chen Hsu, John D. Mountz

**Affiliations:** 1 Division of Clinical Immunology and Rheumatology, the University of Alabama at Birmingham, Birmingham, AL USA; 2 Medicine Service, Birmingham Veterans Affairs Medical Center, Birmingham, AL USA; 3 Division of Clinical Immunology and Rheumatology, King Faisal Specialist Hospital and Research Center, Riyadh, Saudi Arabia; 4 Division of Anatomic Pathology, the University of Alabama at Birmingham, Birmingham, AL USA; 5 Department of Medicine, University of Tennessee Health Science Center, 920 Madison Ave, Memphis, TN 38163 USA

**Keywords:** Systemic lupus erythematosus, Lupus nephritis, B cell interferon beta, Autoantibodies

## Abstract

**Background:**

In systemic lupus erythematosus (SLE), detection of interferon-β (IFNβ) in B cells was found to be most prominent in patients with high anti-Smith (Sm) and renal disease, but a mechanistic connection was not clear. The objective of the present study is to determine the association of IFNβ in peripheral blood naïve B cells with the histopathological features of lupus nephritis (LN).

**Methods:**

The percentage of IFNβ^+^ cells in IgD^+^CD27^−^ naïve CD19^+^ B cells (B cell IFNβ) among peripheral blood mononuclear cells (PBMCs) from 80 SLE patients were analyzed using flow cytometry. Serological and clinical data were collected. The correlations of B cell IFNβ with LN classification and with histopathological findings (light, electron, and immunofluorescence [IF] microscopic analyses for deposition of IgM, IgG, IgA, C1q, and C3) were determined in 23 available biopsy specimens.

**Results:**

B cell IFNβ is positively associated with anti-Sm (*p* = 0.001), anti-DNA (*p* = 0.013), and LN (*p* < 0.001) but was negatively associated with oral/nasal ulcer (*p* = 0.003) and photosensitivity (*p* = 0.045). B cell IFNβ positively correlated with immune complex (IC) deposit in the glomerular basement membrane (GBM) (*p* = 0.002) but not in the mesangial (*p* = 0.107) or tubular region (*p* = 0.313). Patients with high B cell IFNβ had statistically increased development of the proliferative LN (Classes III, IV and/or V), compared to patients with low B cell IFNβ (*p* < 0.0001). Histopathological features positively associated with increased B cell IFNβ included active glomerular lesions as determined by fibrocellular crescents (*p* = 0.023), chronic glomerular lesions indicated by segmental sclerosis (*p* = 0.033), and a membranous pattern of renal damage indicated by spike/holes (*p* = 0.015).

**Conclusion:**

B cell IFNβ correlates with history of severe LN, glomerular basement membrane (GBM) IC deposition, and anatomical features of both active and chronic glomerular lesions.

## Background

Lupus nephritis (LN) results from inflammation through both the innate and adaptive immune responses [1–3]. As with the majority of immune responses, LN is most likely initiated by defects in the innate immune system [1, 2]. Supporting this, dysregulations of the type I interferon (IFN) system and related genes (type I interferon stimulated genes—ISG) were identified as being associated with the development of LN in systemic lupus erythematosus (SLE) [4–7]. DNA and RNA-protein complexes released from apoptotic or necrotic cells can be taken up by phagocytic cells such as the plasmacytoid dendritic cells (pDCs) to produce type I IFN [8, 9]. Type I IFN receptors 1 and 2, which are present on most immune cells as well as parenchymal cells in the target organ of SLE, such as the kidney, lung, vasculature, and skin, can all undergo an inflammatory response after stimulation through the type I IFN receptor complex [10].

The adaptive immune response and the development of autoantibodies (autoAbs) have long been recognized as the cardinal feature of SLE [9, 11–13]. The same DNA and RNA-protein complexes that promote the innate immune response can trigger the development of B cells producing autoAbs against these self-components [3]. We have shown that type I IFN can be produced by and can act upon ribonuclear protein (RNP) autoreactive B cells [14]. Production of autoAbs and certain RNP, such as anti-Smith (Sm), anti-SSA and anti-SSB, have been shown to be a “fixed” feature/value of SLE, whereas other autoAbs, such as anti-DNA vary over time and reflect the activity of disease [15]. We have shown that B cell intracellular interferon beta (IFNβ) correlates most strongly with anti-Sm and that the ability to produce type I IFN by B cells can occur in transitional, naïve, and memory B cells of SLE patients, especially patients who were historically positive with LN [14]. In a mouse model of SLE, the intrinsic production of IFNβ by B cells does not depend upon the environment and is a fixed feature of enhanced TLR7 signaling and the development of autoreactive B cells [16]. Based on the strong correlation of B cell IFNβ with serum anti-Sm and historic positivity of LN in SLE, we correlated IFNβ in naïve B cells in the peripheral blood mononuclear cell (PBMC) with histopathological features of renal biopsy specimens obtained from the same SLE patients. Our results suggest that high B cell IFNβ in SLE patients is highly associated with LN and serological correlates of LN.  Higher B cell IFNβ was associated with immune complex (IC) deposition in the glomerular basement membrane (GBM) and anatomical features of both acute and chronic glomerular lesions.

## Methods

### Study design

This is a retrospective cohort study to examine the correlation of the percentage of IFNβ positive naïve B cells (B cell IFNβ) among SLE and LN patients and compare the clinic-pathologic features and outcomes of high compared to low B cell IFNβ. All medical records were reviewed at the University of Alabama at Birmingham (UAB). Ethical approval for this study was obtained from the ethics committee at UAB.

### Study population

All patients satisfied the following inclusion criteria: age of onset ≥ 18 years and confirmed SLE diagnosis by either the American College of Rheumatology (ACR) 1997 revised criteria [17, 18] or the 2012 Systemic Lupus International Collaborating Clinics (SLICC) classification criteria [19]. The exclusion criteria were as follows: age < 18 years, diagnosis of overlap syndrome, mixed connective tissue disease, or other autoimmune diseases. The studies were conducted in compliance with the Helsinki Declaration and approved by the UAB Institutional Review Board. All data were collected in a manner blinded to IFNβ status until data collection was completed.

### Clinical data collection

Retrospective chart review and data collection were carried out at the time of enrollment visit at the same time as B cell IFNβ measurement. Demographic data included age, race, and sex. Clinical data included age at the time of diagnosis of SLE/LN, mucocutaneous manifestations of malar rash, discoid rash, photosensitivity, oral/nasal ulcer, arthritis, serositis, lupus nephritis, neurological disorder, and hematologic manifestations of leukopenia, hemolytic anemia, and thrombocytopenia. In addition, systemic lupus erythematosus disease activity index (SLEDAI) score, medications used, lupus nephritis class, and laboratory data (serum creatinine level, urine protein/creatinine ratio, anti-DNA and anti-Sm antibodies, C3, and C4) were collected. SLEDAI was used to assess SLE severity at time of enrollment visit based on (1) no activity (score: zero), (2) mild activity (score: 1–5), (3) moderate activity (score: 6–10), (4) high activity (score: 11–19), and (4) very high activity (score: ≥ 20) [20]. SLE duration was defined as the time between the diagnosis of SLE and enrollment into the study. All clinical variables were recorded as present or absent.

### Blood collection for B cell IFNβ, IgG anti-Sm, and anti-DNA analyses

Blood samples were collected from patients on the day of enrollment and were evaluated for B cell IFNβ, circulating IgG anti-Sm antibodies, and anti-DNA (IgM, IgG) antibodies. PBMCs were isolated by density gradient centrifugation (Lymphoprep/SepMate, StemCell Technologies) [15].

### Flow cytometry enumeration of intracellular IFNβ in naïve B cells

Human antibodies included BioLegend BV650-anti-CD27 (O323), PE-Dazzle594-anti-CD19 (HIB19), PerCP-Cy5.5-anti-CD38 (HB-7), BV605 anti-IgD (IA6-2), and PBL Assay Science FITC-anti-IFNβ (MMHB-3). Dead cells were excluded from analysis using the Fixable APC-eFluor® 780 Organic Viability Dye (ThermoFisher) as we previously described [15].

For determining intracellular IFNβ, cells were stained with ef780 viability dye and surface marker fluorochrome-conjugated antibodies, followed by 2% paraformaldehyde (PFA) fixation and 70% ice-cold methanol permeabilization prior to intracellular IFNβ staining.

Flow cytometry data were acquired using an LSRII FACS analyzer (BD Biosciences) and analyzed with FlowJo software (Tree Star Ashland, OR).

### Enzyme-linked immunosorbent assay (ELISA) analysis of autoAbs

Plasma levels of IgG anti-Sm were determined using a commercially available ELISA kit (Alpco, Salem, NH) [15]. Anti-DNA levels were measured using an established protocol [21]. The reaction was stopped with acidification, and the plate was subsequently read at 450–650 nm using an Emax Precision Microplate Reader (Molecular Device, Sunnyvale, CA, USA).

### Renal biopsy

Renal biopsies were evaluated by a renal pathologist in a blinded manner. The renal biopsies were classified according to the ISN/RPS (2004) classification for LN, which is based on the extent of glomerular involvement by light microscopy and whether the injury pattern reflects active lesions (endocapillary hypercellularity, neutrophils/karyorrhexis, fibrinoid necrosis, wire-loop lesions, and cellular/fibrocellular crescents) or chronic lesions (global/segmental glomerulosclerosis and fibrous crescents/broad-based adhesions) [22, 23]. Based on the light microscopic findings, LN was classified as follows: minimal mesangial LN (class I), mesangial proliferative LN (class II), focal LN (class III), diffuse LN (class IV), membranous LN (class V), and advanced sclerosing LN (class VI) [22, 23].

In addition to active (A) and chronic (C) lesions, the glomerular lesions were characterized as either segmental (< 50% glomerular capillary tuft involvement) or global (≥ 50% glomerular capillary tuft involvement) [23, 24]. The activity and chronicity indices were assessed based on the revised ISN/RPS classification for LN, and in addition to the glomerular lesions included tubulointerstitial inflammation and fibrosis [23]. The disease activity index, based on the presence of active lesions, scored mild (score: < 6), moderate (score: 6–12), and severe (score: 12–24). Similarly, the chronicity index, based on the presence of chronic lesions, scored mild (score: < 3), moderate (score: 3–6), and severe (score: > 6–12).

Each renal biopsy was analyzed by immunofluorescence for immune complex deposits, using antisera for IgG, IgA, IgM, C3, C1q, Kappa light chain, and Lambda light chain. The intensity of immune complex deposits, determined by immunofluorescence, was graded with a score of 0–4+ for each of the anatomical regions (mesangium, glomerular basement membrane, tubular basement membrane, arteries/arterioles), with score 0 representing no staining for immune complexes and score 4+ being the highest.

### Statistical analysis

Statistical analysis was carried out using IBM© SPSS© Statistics version 27 (IBM© Corp., Armonk, NY, USA), and the figures were created using GraphPad Prism 9 software (La Jolla, CA). Principal component analysis (PCA) was used to evaluate IFNβ data with clinical characteristics of SLE. Numerical data are expressed as mean and standard deviation or median and range, as appropriate. Qualitative data are expressed as frequency and percentage. Comparison between two groups was made using the Mann-Whitney test or unpaired *t*-test (two-tailed). A chi-square test (Fisher’s exact test) was used to determine differences in the distribution of a categorical variable. A *p*-value < 0.05 was considered significant.

## Results

### Patient characteristics

A total of eighty patients were included in our cohort (Table 1). The mean age at SLE diagnosis was 31.66 years (± 12.82), the mean age at the time of enrollment was 41.21 years (± 13.32), the mean time between the SLE diagnosis and enrollments to study is 9.93 (± 6.71) years, and the mean time between the renal biopsy obtained and B cell IFNβ measurements was 6.63 (± 1.16) years. Sixty-nine percent (*n* = 55) of the patients were African American, 29% (*n* = 23) Caucasian, and 2% (*n* = 2) Hispanic or Latino. Ninety-five percent (*n* = 76) were female. The most prevalent clinical features of the SLE patients in our cohort were arthritis in 68% (*n* = 54), photosensitivity rash in 48% (*n* = 38), LN in 41% (*n* = 33), and oral/nasal ulcer in 40% (*n* = 32). Among the 33 subjects with LN, 58% (*n* = 19) had proliferative LN either isolated or combined with class II or V. Seventy-four percent (*n* = 59) had active disease (SLEDAI score: ≥ 1) at the time of enrollment. At this time, 49% (*n* = 39) had mild (SLEDAI score: 1–5), 20% (*n* = 16) had moderate (SLEDAI score: 6–10), 1% (*n* = 1) had high (SLEDAI score: 11–19), and 4% (*n* = 3) had very high activity (SLEDAI score: ≥ 20). Eighty-nine percent (*n* = 71) were on hydroxychloroquine or quinacrine or both and 51% (*n* = 41) were on prednisone at the time of enrollment. The mean anti-DNA-IgG (OD), anti-Sm (unit/ml) and IFNβ+ naïve B cells (%) at the time of enrollment were 0.34 (± 0.16), 55 (± 75.53), and 46 (± 29.40), respectively. Sixteen healthy donors were included at the same time.

**Table 1:** Baseline clinical characteristics of the participants at the time of enrollment

Characteristics	Total patients (***N*** = 80, 100%)
Age at time of enrollment-year, mean (SD)	41.21 (± 13.32)
Age at time of diagnosis of SLE-year, mean (SD)	31.66 (± 12.82)
SLE duration-year, mean (SD)	9.93 (± 6.71)
Race	
Caucasian	23 (29)
African American	55 (69)
Hispanic or Latino	2 (2)
Sex	
Female	76 (95)
Male	4 (5)
Clinical features	
Mucocutaneous disorder	
Malar rash	27 (34)
Discoid rash	12 (15)
Photosensitivity	38 (48)
Oral/nasal ulcers	32 (40)
Arthritis	54 (68)
Serositis (pleuritic, pericarditis)	18 (23)
Lupus nephritis	33 (41)
Autoimmune hepatitis	1 (1)
Pancreatitis	1 (1)
Cardiomyopathy	1 (1)
ILD	1 (1)
Neurological disorder (psychosis, seizure, CNS vasculitis)	7 (9)
Hematological disorder	
Hemolytic anemia	4 (5)
Leukopenia	20 (25)
Thrombocytopenia	16 (20)
SLEDAI (score)	
No activity (0)	18 (22)
Mild (1–5)	39 (49)
Moderate (6–10)	16 (20)
High activity (11–19)	1 (1)
Very high activity (≥ 20)	3 (4)
Missing, (%)	3 (4)
Medication at time of enrollment	
Hydroxychloroquine, Quinacrine or both	71 (89)
Prednisone	41 (51)
MTX or leflunomide	19 (24)
MMF, Myfortic or AZA	19 (24)
Belimumab	11 (14)
Immunology laboratory results	
Anti-DNA (titer), mean (SD)	36 (± 58.13)
Anti-DNA- IgM (OD), mean (SD)	0.24 (± 0.12)
Anti-DNA- IgG (OD) mean (SD)	0.34 (± 0.16)
Anti-Smith (unit/mL), mean (SD)	55 (± 75.53)
Anti-Smith (unit) at any time, mean (SD)	68.7 (± 143.78)
C3 (mg/dL), mean (SD)	118 (± 32.40)
C4 (mg/dL), mean (SD)	23 (± 11.22)
% of IFNβ-naïve B cells, mean (SD)	46 (± 29.40)
Lupus nephritis class	
Class I	1 (1)
Class II	9 (11)
Class III	5 (6)
Class IV	6 (7)
Class V	4 (5)
Class II and III	1 (1)
Class V and III	5 (6)
Class V and IV	2 (2)

*SLE* systemic lupus erythematosus, *ILD* interstitial lung disease, *CNS* central nervous system, *SLEDAI* Systemic Lupus Erythematosus Disease Activity Index, *HCQ* hydroxychloroquine, *MTX* methotrexate, *AZA* azathioprine, *MMF* mycophenolate mofetil, *Myfortic* mycophenolic acid

### B cell IFNβ in lupus and control groups

**Fig. 1:**
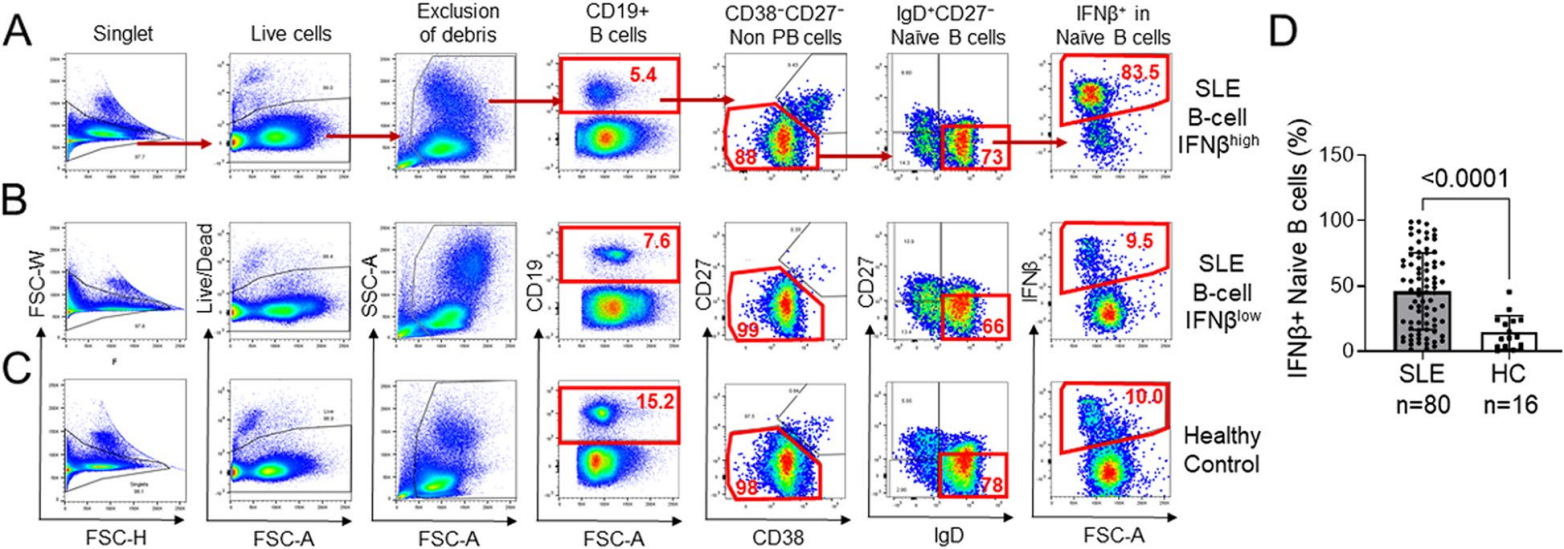
Flow cytometry analysis of IFNβ^+^ naïve B cells. **A**–**C** Flow cytometry plots showing the sequential gating of IFNβ in naïve B cells based on the indicated cell surface markers in a representative SLE patient with high B cell IFNβ (**A**), a representative SLE patient with low B cell IFNβ (**B**), and a representative healthy control donor (**C**). **D** Bar graph showing the percentages of IFNβ^+^ naïve B cells in SLE patients and healthy controls (PB, plasmablasts/plasma cells, HC; healthy control) (data are mean ± SD; unpaired *t*-test)

The flow cytometry gating strategy used to determine the percent of IFNβ positive IgD^+^CD27^−^CD38^lo^CD27^lo^ subset of CD19+ naïve B cells is shown in Fig. 1A–C. There was a statistically significant increase in B cell IFNβ of SLE patients (*n* = 80; mean 46.0 ± 29.4) compared to healthy controls (*n* = 16; mean 14.04 ± 13.04; *p* < 0.0001) (Fig. 1D).

### Association of B cell IFNβ with SLE clinical features

**Fig. 2:**
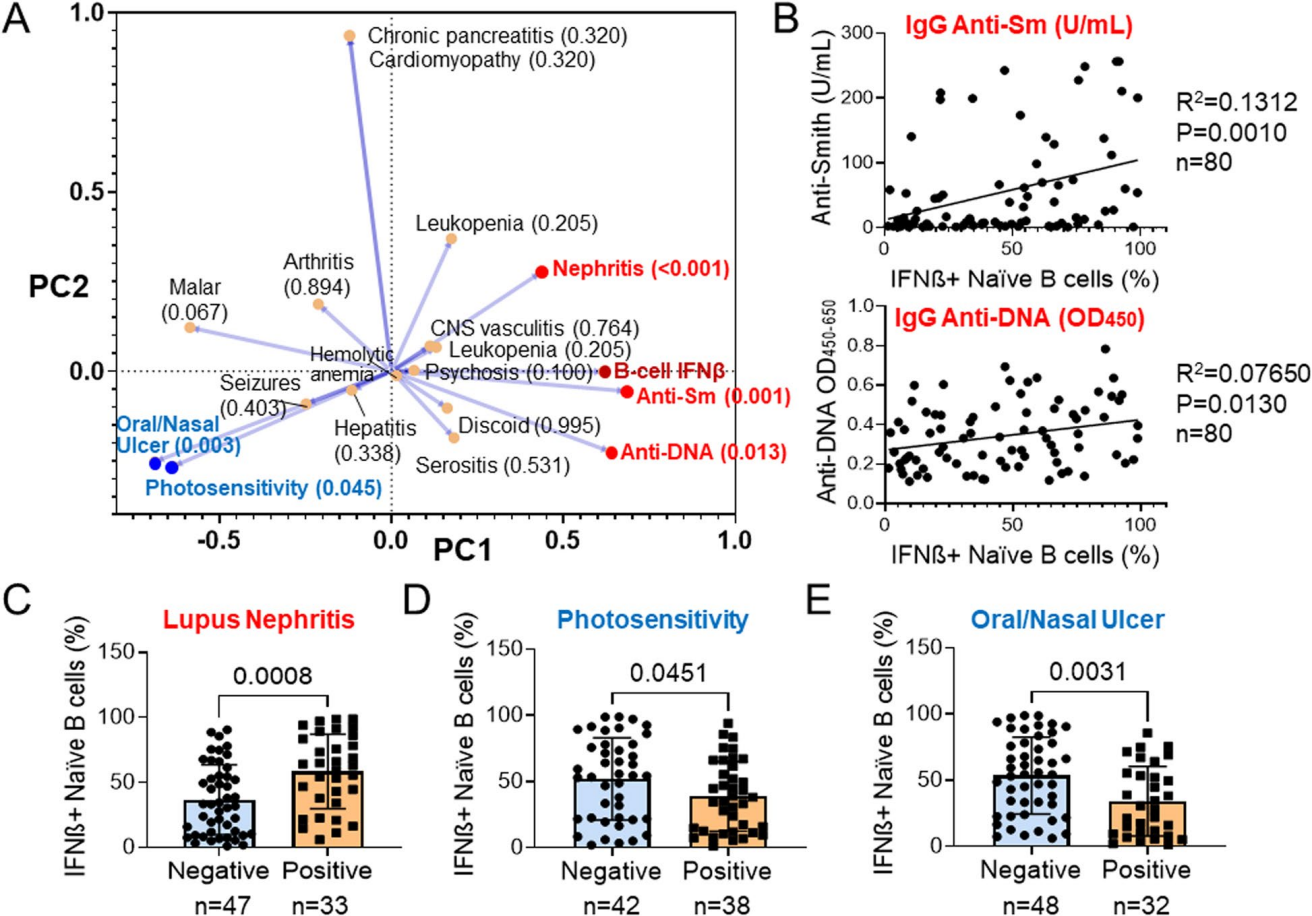
Association of clinical and laboratory parameters with B cell IFNβ in SLE patients. Eighty SLE patients were analyzed for B cell IFNβ and other clinical and laboratory characteristics. **A** Principal component (PC) analysis of 18 parameters and B cell IFNβ. The clinical parameters indicated near the position of the PC analysis plot and the *p* value of each parameter as being associated with B cell IFNβ is shown in the parenthesis. Variants shown in red exhibit significant positive correlation with B cell IFNβ. Variants shown in blue exhibit significant negative correlation with B cell IFNβ. **B** Linear regression analysis of anti-Smith (top) and anti-DNA (bottom) with B cell IFNβ. Anti-Sm was shown as U/mL. Anti-DNA was shown as OD_450_. *R*^2^, *p* value, and number of subjects (*n*) are indicated. **C**–**E** Bar graph showing the percentages of IFNβ^+^ naïve B cells in SLE patients with (positive) or without (negative) LN (**C**), photosensitivity (**D**), and oral/nasal ulcer (**E**) (mean ± SD; unpaired *t*-test with the *p* value shown in each panel)

Clinical and laboratory features of SLE in 80 patients, along with B cell IFNβ, were analyzed by principal component analysis (PCA) (Fig. 2). The percentage of IFNβ+ naïve B cells was significantly correlated with anti-Sm (*p* = 0.001) and anti-DNA (*p* = 0.013) (Fig. 2A, B). Patients with nephritis (*p* < 0.001) exhibited a significantly elevated B cell IFNβ (Fig. 2A, C). In contrast, patients with photosensitivity (*p* = 0.045) or oral/nasal ulcers (*p* = 0.003) exhibited a significantly lower B cell IFNβ (Fig. 2A, D, E).

### Association of B cell IFNβ with clinical indicators of LN

**Fig. 3:**
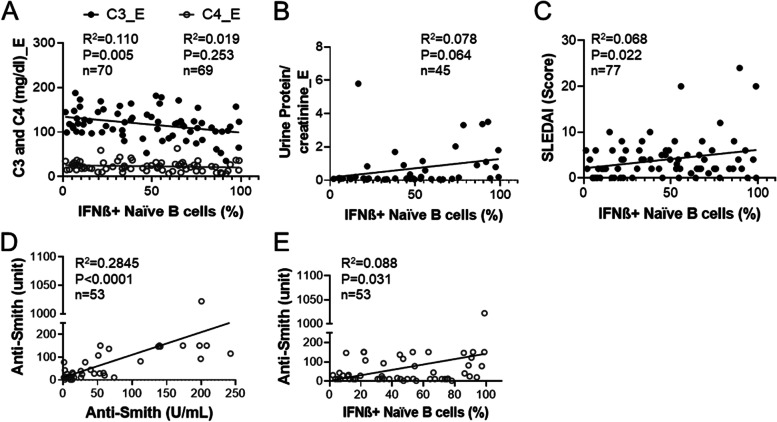
Association of B cell IFNβ with LN-related laboratory parameters and SLEDAI. **A** Complement C3 (mg/dL) and C4 (mg/dL) at the time of enrollment were plotted against the percentages of IFNβ^+^ naïve B cells. **B** Urinary protein/creatinine ratio at the time of enrollment was plotted against the percentages of IFNβ^+^ naïve B cells. **C** SLEDAI score was plotted against the percentages of IFNβ^+^ naïve B cells. **D** Anti-Sm at any time was plotted against anti-Sm at the time of enrollment. **E** Anti-Sm (unit) at any time was plotted against the percentages of IFNβ^+^ naïve B cells (linear regression analysis. The *R*^2^, *p* value, and the number of subjects are shown on the plot)

Other laboratory indicators of active disease or renal disease, including C3, C4, and urine protein/creatinine ratio, were reviewed/examined at the time of B cell IFNβ analysis. There was a negative correlation between B cell IFNβ and diminished levels of C3 but not C4 (*p* = 0.005, *p* = 0.253, respectively) (Fig. 3A). There was a near significant correlation between the percentage of B cell IFNβ with urine protein/creatinine ratio at the time of enrollment (*p* = 0.064) (Fig. 3B). There was a significant correlation between the B cell IFNβ and the SLEDAI score at the time of enrollment (*p* = 0.022) (Fig. 3C).

The titer of anti-Sm autoAb has been shown previously to not vary over time in SLE patients [25]. Among the 80 patients, clinical lab measurement of anti-Sm levels at the time near biopsy was available for 53 patients. Consistent with this, there was a highly significant correlation between the levels of anti-Sm near the time of biopsy and at the time of patient enrollment (*p* < 0.0001) (Fig. 3D). There was also a significant correlation between the anti-Sm levels at any time and B cell-IFNβ for these 53 subjects (*p* = 0.031) (Fig. 3E). These results suggest that B cell IFNβ may be a fixed feature in LN patients.

### Kidney IC in patients with high or low B cell IFNβ

**Fig. 4:**
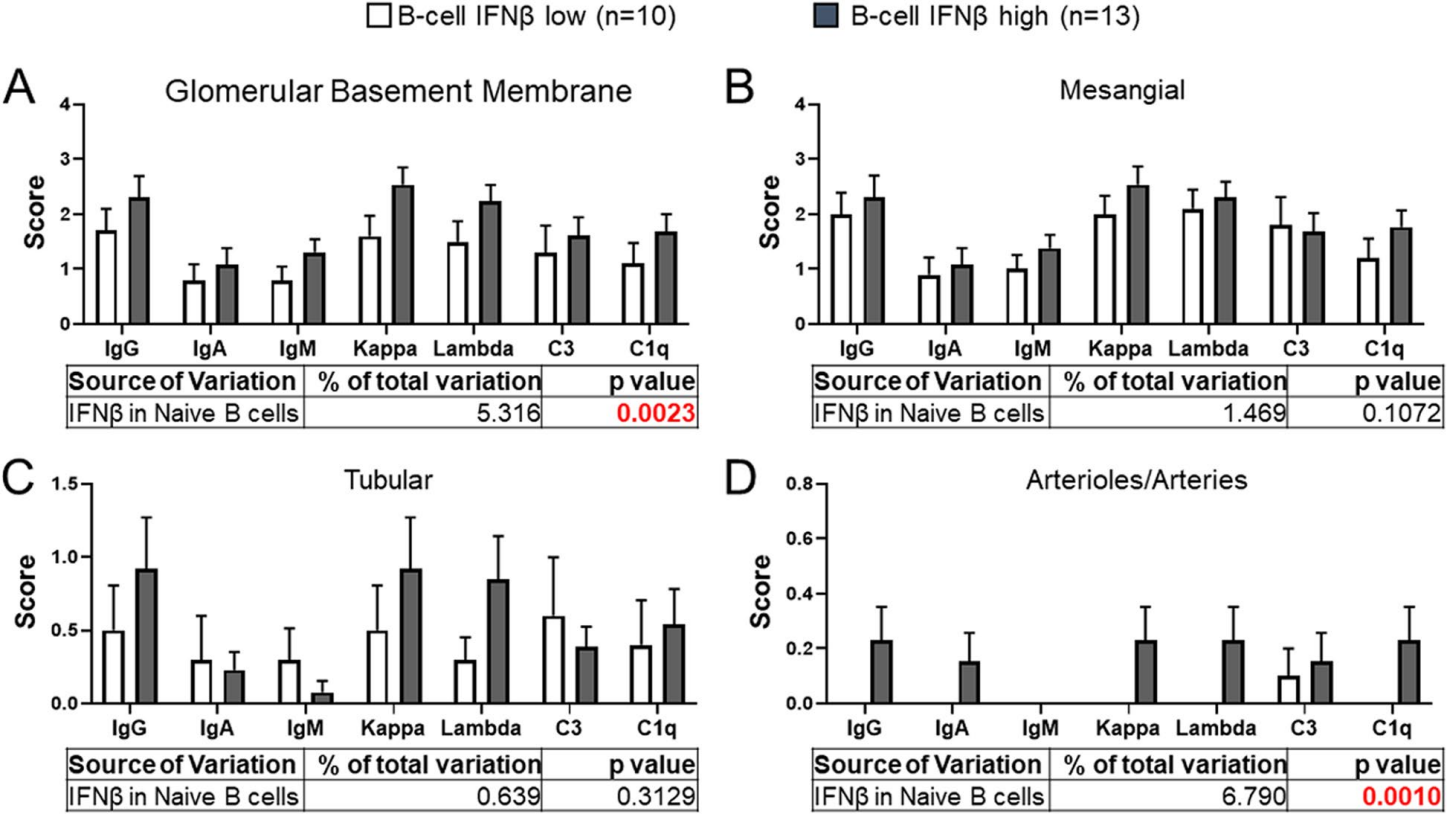
Association of B cell IFNβ with the location of immune complexes in renal biopsy. Renal biopsies were obtained from 23 available specimens. The immune complexes were scored based on the presence each indicated type in the glomerular basement membrane (**A**), mesangium (**B**), renal tubular (**C**), and arterioles/arteries (**D**) using a 0 to 4 scoring range: 0, absent, 1 rare, 2 low abundance, 3 moderate abundance, and 4 high abundance. LN patients were divided into two groups based on the levels of B cell IFNβ either below the mean (IFNβ low, *n* = 10) or above the mean (IFNβ high, *n* = 13) (data are mean ± SEM; 2-way ANOVA test on the effects of B cell IFNβ and IC type in each region)

Immune complex deposition can be identified in the GBM, mesangium, tubular region, or renal artery in LN [26]. We next determined if B cell IFNβ could be associated with renal IC deposition. Renal biopsy specimens were obtained from 23 available specimens among the 80 SLE patients. SLE patients were divided into two groups, determined by having B cell IFNβ below the mean (IFNβ low, *n* = 10) and above the mean (IFNβ high, *n* = 13). The staining for all the immunoreactants i.e., IgG, IgA, IgM, Kappa, Lambda, C3, and C1q was increased along the GBM in the biopsy of patients with high B cell IFNβ, compared to patients with low B cell IFNβ (*p* = 0.002), indicating increased deposition of IC in the former category of the lupus patients Fig. 4A. Although the IC in the mesangium and tubules of the biopsy of the high B cell IFNβ group was more prevalent compared to the low B cell IFNβ group; this did not reach statistical significance (*p* = 0.107, *p* = 0.313, respectively) (Fig. 4B, C). IC deposition was rare in the arterioles/arteries in our cohort of lupus patients, but when observed, it was almost exclusively present in the high B cell IFNβ subgroup and was statistically significant (*p* = 0.001) (Fig. 4D).

We concluded that increased IC deposits along the GBM of the high B cell IFNβ LN patients compared to low B cell IFNβ LN patients is consistent with the finding that B cell IFNβ positively correlates with anti-Sm (Fig. 2A, B) at the time of enrollment and also at any time (Fig. 3E).

### Association of B cell IFNβ with the classification of LN

**Fig. 5:**
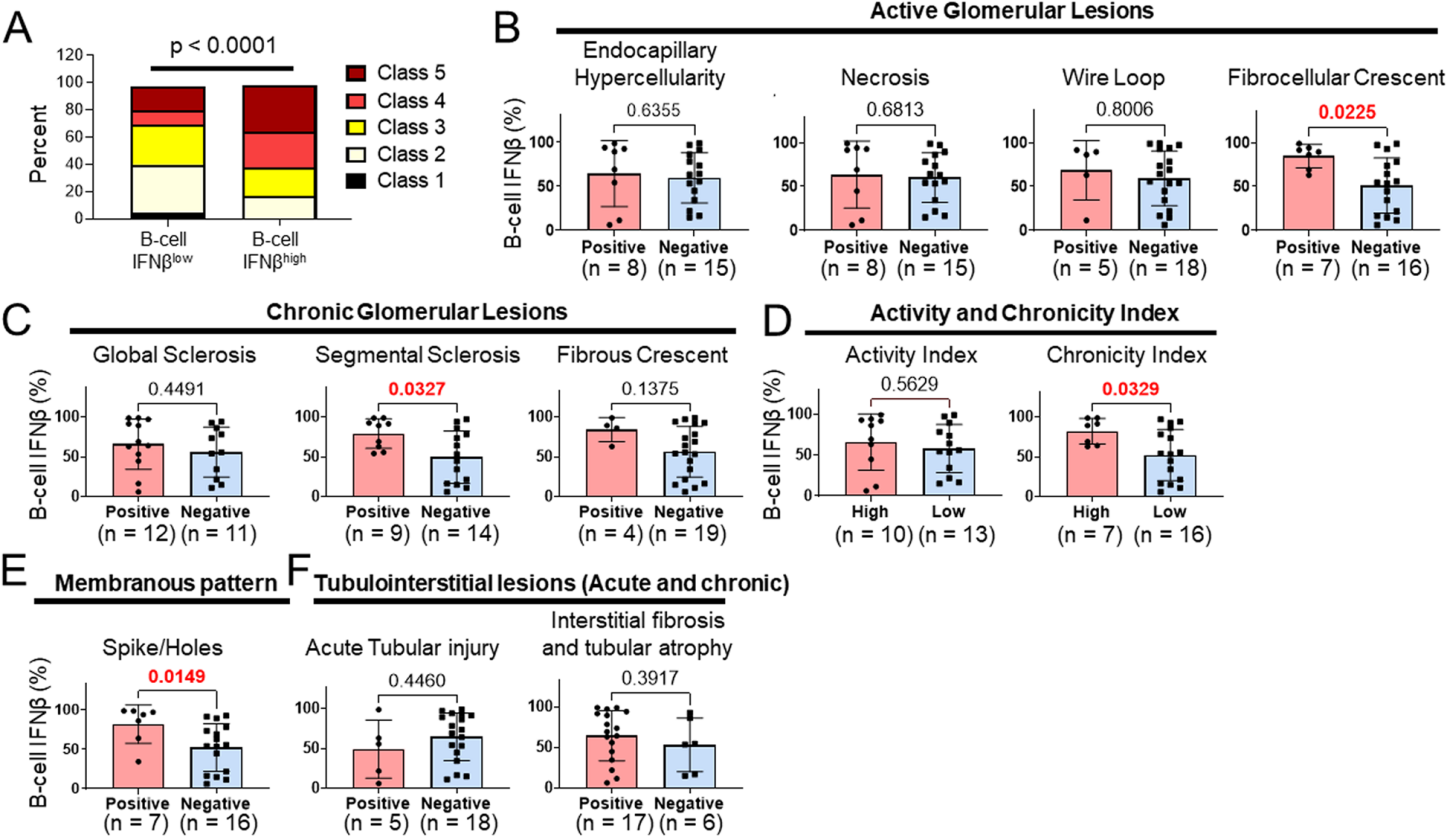
Quantitative analysis of renal biopsy features with B cell IFNβ. **A** The distribution of LN classification in patients with low (lower 50%) versus high (upper 50%) B cell IFNβ. The *p* value (chi-squared test) is shown above the plot (*n* = 33). **B**–**F** The histologic features were analyzed using renal biopsy specimens available from 23 SLE patients. For each histologic feature, the individual percentage of IFNβ^+^ naïve B cells was plotted for each subject and indicated by the data-point on the graph, ranging from 0 to 100%. Histologic features associated with active glomerular lesions (**B**), chronic glomerular lesions (**C**), activity index and chronicity index (high: above the mean; low: below the mean) (**D**), membranous patterns of spike/holes (**E**), and tubulointerstitial lesions (**F**). The *p* value for each comparison is indicated as the number above the groups. Statistically significant differences are indicated in red. (data are mean ± SD; two-tailed Mann-Whitney test)

To determine whether LN classification correlated with B cell IFNβ, we compared LN classification from the available 33 SLE patients in our cohort. Patients in the high B cell IFNβ group exhibited a greater tendency to develop class III and IV +/− class V LN compared to patients in the low B cell IFNβ group (*p* < 0.0001) (Fig. 5A). Overall, these results suggest that the higher percentages of IFNβ+ naïve B cells are observed in the patients who historically have developed more severe renal disease.

### Association of B cell IFNβ with histopathologic features of LN

LN histopathological features were further analyzed from 23 available specimens from the present cohort. The histology slides were not available for 10 patients, as the biopsies were performed outside the present institution.

A comprehensive analysis was carried out to determine the association of the presence or absence of 12 different histologic features at the time of biopsy with B cell IFNβ (Fig. 5B, C, D, E, F). These histologic features were divided into active glomerular and chronic glomerular lesions [23]. There was a significant difference in B cell IFNβ in LN patients with or without fibrocellular crescents, segmental sclerosis, and chronicity index (*p* = 0.023, *p* = 0.033, *p* = 0.033, respectively) (Fig. 5B, C, D). In addition, we found that the membranous pattern manifested as spikes and holes by light microscopy, was statistically significant (*p* = 0.015) (Fig. 5E). There was no significant difference in B cell IFNβ in patients with or without tubulointerstitial lesions, acute tubular injury, or interstitial fibrosis and tubular atrophy (Fig. 5F).

## Discussion

LN has been shown to be strongly correlated with anti-Sm [15]. The results suggest that B cell IFNβ is most strongly associated with the levels of anti-Sm that have been found to be stable over time. The present findings suggest that B cell IFNβ may be one factor underlying the development of anti-Sm which in turn leads to renal disease by forming IC, direct binding to Sm or other autoantigens in the kidney, as previously proposed [14]. Alternatively, we have shown that expression of IFNβ in naïve B cells is strongly correlated with intracellular IFNβ detected in all subpopulations of B cells, including transitional B cells, memory B cells, as well as in pDCs in PBMC [15]. Previous investigators have shown that type I IFN produced by tubular cells in the kidney is associated with LN [27]. Another possibility is that IFNβ produced in B cells reflects the ability of IFNβ produced in other cells such as renal tubular cells. We are currently analyzing this possibility in renal biopsy samples from SLE patients.

The present results are consistent with previous findings showing that elevated type I IFN stimulated genes and the development of lupus nephritis were the most prevalent in a population of SLE patients with African ancestry [28]. Jenks et al. showed that there was a disproportionally higher number of African American SLE who developed active disease, nephritis, high anti-Smith, and anti-RNP autoAbs. These patients also developed an increased population of activated naïve and double-negative 2 B cells as a consequence of abnormal TLR7 stimulation [29]. We previously showed that in the BXD2 mouse model of lupus, transitional B cell endogenous IFNβ was the initial step enabling assembly and efficient signaling through TLR7 [30]. This lead to B cell activation and survival with up-regulation of CD69 and CD86 on naïve B cells [30].

The mechanism of LN by these antibodies is through their ability to form IC that can be found in the glomerulus, interstitium of the kidney or by binding to cross-reactive antigenic targets in the kidney [31]. The primary function of complement component C3 in prevention of IC-mediated disease in SLE is its ability to break down or diminish the size of ICs by displacing autoAbs and its subsequent function in enhancing uptake of ICs by phagocytic cells that can degrade these complexes [3]. It has been proposed that low levels of C3 are one of the main factors that lead to IC formation and IC localization in the kidney [32]. Also, genetically determined deficiencies of C3 are well known predisposing factors for SLE, but more recently, genetically determined factors leading to modestly low levels of C3 have been proposed as a factor leading to IC in renal disease in SLE [33]. We propose that the combined production of autoAbs, resulting in part from high B cell IFNβ, and low C3 complement levels, also genetically determined, can together predispose to LN.

Type I IFN has previously been found to mediate LN from the initial stages to the development of renal fibrosis [27, 34]. At the initial stages, type I IFN contributes to formation of IC as well as decreased clearance of ICs [35]. It has been shown that renal resident cells rather than infiltrating immune cells are a main source of type I IFN [34] and can be stimulated through TLR by way of nucleic acid products including DNA and RNA-immune complexes which can induce IFNβ in renal medullary cells (RMC) [9, 36]. Thus, IFNβ can have a dual factor of inducing IC, as well as decreasing clearance of ICs at the acute stage.

The present results also suggest that high type I IFN promotes chronic changes such as glomerular sclerosis [37, 38]. Previous results have shown that IFNβ can stimulate podocyte actin B-1 expression and actin remodeling [39] while IFNα has been shown to accelerate glomerular epithelial cells dysfunction and cause epithelial cell apoptosis which increases glomerular epithelial cell permeability [40]. Type I IFN also has been shown to promote renal medullary cells and lead to glomerular fibrosis through hyperplasia and proliferation through the induction of metalloproteinases and growth factors [41]. We previously showed that type I IFN can enhance “stiffness” of macrophages [42]. In the kidney, development of renal myofibroblasts which leads to excessive accumulation of extracellular matrix is a common feature of chronic renal disease [43, 44]. The pathogenicity of type I IFN in LN therefore can be multifaceted. Indeed, the present results indicated that patients with more severe LN also exhibited higher B cell IFNβ in the periphery.

The current studies are based on an in-depth chart review in assessing all the clinical parameters of SLE. We now identified that although B cell IFNβ correlated with history of lupus nephritis, it negatively correlated with photosensitivity and oral/nasal ulcer in SLE. The present unsupervised comprehensive analysis of organ-associated lesions in 80 SLE patients is in agreement with results by others that patients with active discoid lupus rarely exhibit progressive renal disease [45–47]. These results are also consistent with recent findings by others showing that type I IFN activity is highly associated with active LN but has weak association with photosensitivity and mucocutaneous lupus [48]. The same study further shows that type I IFN is highly associated with the development of anti-Sm and ANA but has weak or no association with the development of non-RNP autoantibodies [48]. These results together suggest that patients who developed severe lupus nephritis and anti-Sm may have persistent high percentages of IFNβ^+^ naïve B cells. Also, there are differential immune dysregulations involved in the development of renal disease compared to cutaneous disease in SLE.

The limitation of this study is that it is a retrospective study of LN biopsy specimens, with renal biopsy samples available for only 23 of the enrolled patients. Prospective longitudinal multicenter studies will be needed to determine if B cell IFNβ correlates with current LN and if it predicts the onset or severity of LN. In addition, specific clinical information and labs (urine protein/creatinine ratio, anti-DNA, anti-Sm, and treatment details) were not available for some biopsied patients, and assessments for IFNβ were not contemporaneous with renal biopsy.

## Conclusions

In summary, the results suggest that high percentages of IFNβ^+^ in naïve B cells in SLE patients are associated with severe LN and serological correlates of nephritis, as well as IC deposition and anatomical features of both active and chronic glomerular lesions. These results provide insights into the pathogenic mechanisms of B cell IFNβ concerning the production of autoAbs, immune complexes, and renal disease tissue damage that can help guide future therapies that target type I IFN for the treatment of SLE.

## Data Availability

The datasets used and/or analyzed during the current study are available from the corresponding author on reasonable request.

## Abbreviations

SLE: Systemic lupus erythematosus
IFNβ: Interferon-β
Anti-Sm: Anti-Smith
LN: Lupus nephritis
IF: Immunofluorescence
GBM: Glomerular basement membrane
IC: Immune complex
IFN: Interferon
pDCs: Plasmacytoid dendritic cells
autoAbs: Autoantibodies
RNP: Ribonuclear protein
PBMC: Peripheral blood mononuclear cell
B cell IFNβ: IFNβ positive naïve B cells
UAB: University of Alabama at Birmingham
ACR: American College of Rheumatology
SLICC: Systemic Lupus International Collaborating Clinics
SLEDAI: Systemic lupus erythematosus disease activity index
PFA: Paraformaldehyde
PCA: Principal component analysis
RMC: Renal medullary cells
